# A systematic review of the literature describing the outcomes of near-peer mentoring programs for first year medical students

**DOI:** 10.1186/s12909-018-1195-1

**Published:** 2018-05-08

**Authors:** Olawunmi Akinla, Pamela Hagan, William Atiomo

**Affiliations:** 1 Macclesfield District General Hospital, Macclesfield, UK; 2 School of Medicine, The University of Nottingham, Nottingham, UK; 3 School of Medicine, Queens Medical Centre, The University of Nottingham, Derby Road, Nottingham, NG7 2UH UK

**Keywords:** Peer-mentoring, Near-peer, Outcomes, Evaluation

## Abstract

**Background:**

Transition into higher education has been identified as one of the most stressful periods for learners. Interventions targeting the transition phase such as near- peer mentoring might help address some of these challenges. We were however unable to identify a published systematic review of the literature describing outcomes of near-peer mentoring of medical students during the transition phase into medical school. The aim of this paper is to review the literature and describe the outcomes of near-peer mentoring schemes for first-year medical students in the transition phase.

**Methods:**

A search of different electronic databases was carried out, using the search terms peer, buddy, mentor*, counsel*, advise*, tutor*, student, medical, school. 1861 articles were identified, however only 5 studies met the inclusion criteria**-** primary mentees should be first-years, and mentors must be inclusive of second-years but not limited to them. In reporting this paper, the PRISMA guidelines were followed.

**Results:**

Published material on near-peer mentoring for medical students is scarce. Three outcomes for peer mentoring were identified- professional and personal development, stress reduction, and ease of transitioning. Incidentally, peer-mentoring was also found to have facilitated the development of personal and professional attitudes in the mentors. The quality of the evaluation methods in the studies was however low to moderate.

**Conclusion:**

Near-peer-mentoring is a way of promoting professional and personal development. It is also promising to aid transition and maintain well-being of first-year medical students. However, larger, better quality longitudinal studies, are needed to ascertain its true value for these students.

## Background

The earliest known use of the term ‘mentor’ is in Greek mythology where Athena disguised herself as Mentor for the purpose of looking after Odysseus’ son Telemachus while Odysseus sailed against Troy [1]. Peer mentoring has however more recently been defined as a formal relationship in which a more qualified student provides guidance and support to another student [1]. It is a method used by different universities to support students in acclimatising to the new University environment [2]. The support of new students is important to address the diversity of students and the resulting problems which may arise during the introduction to their tertiary studies [3]. It is also important to reduce attrition rates, especially now with the use of performance indicators to rate universities. Furthermore, greater responsibility has been placed in the hands of universities to ensure student success [4]. Universities now have the responsibility to widen access into their institutions and peer- mentoring may help [4, 5]. The cost of attending university is high, and as such justifications must be made for this expense [4, 6].

A near-peer is one who is one or more years senior to another on the same level of education training, that is, learners providing pastoral support to other learners in contrast to faculty staff mentoring learners [7]. As a result, a near-peer mentoring relationship may be defined as one in which a more senior learner (a year or more above) provides guidance and support to a new junior learner to enable the new student to navigate his or her education.

Kram [1] divides the function of mentoring into two; task/ career related and psychosocial functions. Other authors on mentoring have also added role modelling as a third function [8]. Individual studies on peer mentoring in the nursing and biomedical disciples suggest a positive benefit [9–11], particularly with transition into higher education [12]. Transition into higher education has been identified as one of the most stressful periods for learners. Interventions targeting the transition phase such as near- peer mentoring might help address some of these challenges [13]. It has been suggested that a near-peer mentor, being close to the social, professional, or age level of the new learner, may enhance his or her cognitive and psychomotor development [14].

We were however unable to identify a published systematic review of the literature describing outcomes of near-peer mentoring of medical students during the transition phase into medical school, although we identified a published protocol [12] for a planned mixed methods systematic review of the effectiveness of peer mentoring in promoting a positive transition to higher education for first-year undergraduate students. The aim of our study was to review the literature and describe the outcomes of near-peer mentoring schemes for first-year medical students in the transition phase into the medical school. It was important to synthesize the literature separate from “peer mentoring” or “peer teaching,” to minimise heterogeneity and confounding variables in analysing and interpreting our results. The peer mentoring literature for example can include faculty members acting as peer mentors.

This gap in the literature was also relevant within our local context at the Nottingham Medical School, where, in response to a University-wide initiative, a near peer mentoring scheme- to strengthen and support the transition phase into medical school- was established in the five-year undergraduate medicine course at the University of Nottingham, United Kingdom in 2014. Our specific objectives were to:

1. identify similar mentoring programmes in medical schools in already published literature,
2. determine how evaluation was carried out in such programmes, and
3. describe the outcomes measured.

## Methods

To begin the search, a hierarchy of evidence [15] was chosen. This helped to structure the search process and identify the relevant papers. It also helped to direct to relevant databases and search engines. The hierarchy of evidence used for this review include:

1. randomised controlled trials
2. systematic reviews
3. quantitative studies
4. qualitative studies/policy documents
5. expert opinion

A search across different electronic databases using different combinations of peer, buddy, mentor*, counsel*, advise*, tutor, medical, student, and school altered as appropriate was carried out. An example of combinations of search terms used was “peer mentor*” AND “medical student”. Complex combinations were not used to prevent limiting the search sensitivity whilst maintaining its precision [16]. The following electronic databases were used: PubMed, Embase, Scopus, ERIC, Ovid, PsycINFO, and the Cochrane Highly Sensitive Search Strategies [16] for identifying randomized control trials in MEDLINE and Ovid. Google Scholar was also searched to identify grey literature. No time restrictions were used, and the search was conducted in October 2017. The references of studies meeting the search criteria were also searched due to the limited number of relevant papers obtained from the initial search. Abstracts of papers were read by the first author to determine if they were relevant to the research question. Twenty-two articles were identified as being relevant and full-text articles of these papers were obtained. Following this, the publications were independently assessed by both the first author, and a colleague, A.C to determine the papers which matched the inclusion and exclusion criteria. Inclusion criteria set were:

1. the programmes should have first-year students as the primary mentees;
2. the mentors must be near-peers i.e. 2nd years but not limited to them.

Papers excluded were near-peer mentoring programmes associated with other interventions such as tutoring and individualised academic planning. This was to keep with the aforementioned definition of peer mentoring as guidance, and to limit variables such as tutoring that could affect the results of the study [17].

**Fig. 1:**
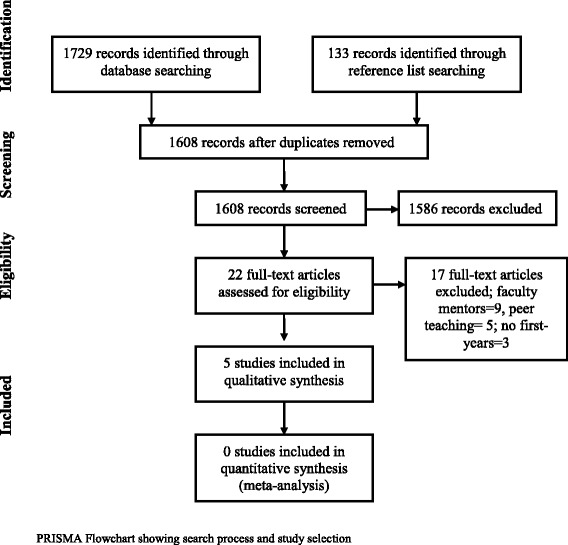
PRISMA Flowchart showing search process and study selection

At the end of this process, five papers were identified as relevant to the study (Fig. 1). In reporting this paper, the Preferred Reporting Items for Systematic Reviews and Meta-Analyses (PRISMA) [18] guidelines were followed. These papers were thematically analysed according to the recommendations by Lincoln and Guba [19]. The findings of each paper were highlighted, and written down. This was done line by line for each study. Data was then abstracted into a Microsoft Excel spreadsheet and findings were compared to each other. This was done to identify similarities between the findings and led to the development of codes. Recurrent codes across the papers were stress reduction, resilience, improving wellbeing, making adjustment easier, support, learning teamwork and respect, time-management, building confidence, learning “tricks of the trade”. The developed codes led to the development of the outcomes identified in this study.

### Critical appraisal of studies

The studies were appraised for quality using the Crowe Critical Appraisal Tool (CCAT) [20]. As at yet, there is no validated critical appraisal tool for assessing the validity and reliability of cross-sectional studies [15]. It was developed based on existing tools, general research methods theory and reporting guidelines. It has reported validity and reliability data better than that of informal appraisal tools. The CCAT can be used for both quantitative and qualitative studies. It has 22 items divided under 8 categories. Each item has multiple item descriptors that make it easier to appraise and score a category. The categories include: Preliminaries, Introduction, Design, Sampling, Data collection, Ethical matters, Results and, Discussion. The categories are scored on a 6 point scale of 0 – 5. The maximum amount of scores that can be gotten from this tool is 40 while the minimum is 0. The scores are converted into a corresponding percentage provided along with the tool. For example, the title, study aims and design of each study were scored based on whether they were appropriate and relevant to the study and abstracts based on whether it was balanced and informative and contained all the key information covered in the research. The introduction/background of each was scored based on whether a description of why the study was undertaken was provided, a summary of current knowledge was present and if there was a link between the stated aims or objectives and the problem identified to be addressed.

Research methods were scored based on whether a rationale for the study design was clearly stated, the appropriateness of the design used to evaluate the study’s question(s), sampling methods used and how the sample size was determined. The rationale of the data collection tool used was appraised, whether a description of this tool was provided, data collection methods and how issues such as sampling bias were dealt with. Studies were also scored for quality based on whether they provided reliability and validity data for the data collection tool used. The limitation of this tool is that it is dependent on the appraiser’s scoring, and there is a temptation to overlook performance in the individual categories and only focus on the total score.

## Results

Five studies [21–25] met the inclusion criteria (Table 1).

**Table 1:** Overview of papers

	AIM OF STUDY	COUNTRY YEAR	PROGRAMME STRUCTURE	PROGRAMME GOAL	STUDY TYPE	DATA COLLECTION TOOL	STUDY FINDINGS
Abdolalizadeh et al. [24]	To explore the perceptions of mentors and mentees of the dual mentoring programme	Iran 2017	One-on-one and group mentoring. Mentor:mentee ratio 1:3	To assist the new incoming first-years in settling in, as well as adjusting to the new outcome-based curriculum	Descriptive	Interviews- qualitative data	a) Mentees had positive perceptions of the dual mentoring programme b) Mentees felt supported in the psychosocial domain c) Mentees felt supported academically c) Mentors reported benefits in their personal development
Singh et al. [22]	a) To evaluate near-peer mentoring of new medical students b) To compare faculty mentoring with near-peer mentoring	India 2014	Group mentoring. Near-peer Mentor:mentee ratio 1:3	To facilitate an easy adaptation of the first years into the medical school	Descriptive	Questionnaire- quantitative and qualitative data obtained	a)Mentees met more often with the near-peer mentors than faculty b)Mentees benefitted academically, socially and emotionally c)Mentors reported improvements in the affective domain, then cognitive and psychomotor domain d) Mentors benefitted from being mentored by faculty members
Yusoff et al. [23]	To evaluate medical students’ perception and attitudes towards the near-peer mentoring programme	Malaysia 2010	Group mentoring. Mentor:mentee ratio not mentioned	To facilitate the 1st-year students’ adjustment to new campus life as well as to promote their personal development	Descriptive	Validated Questionnaire- quantitative data	a) Mentees and mentors had positive perceptions of the programme b) both had benefits in affective domain c)both reported better communication and interpersonal skills
McLean [21]	To evaluate if studying different curricula affected peer mentoring	South Africa 2007	Group mentoring. Mentor:mentee ratio 1:10	To assist in the social, academic and psychological integration of new students into the school	Descriptive	Questionnaire- quantitative and qualitative data obtained	a) Mentees and mentors reported increase in the affective domain. b) both reported better interpersonal skills
Kosoko-Lasaki et al. [20]	To determine the outcome measures of the mentoring programme	USA 2006	One-on-one and group mentoring. Mentor:mentee ratio not mentioned	To increase the opportunities for counselling, mentoring and group support of the minority student population	Descriptive	Questionnaire- quantitative and qualitative data obtained	a) Mentees perceived the programme to be effective b) Mentees reported an increase in their perceived levels of professionalism c)The mentoring programme provided an avenue for peers to interact and build friendships

### Methods of evaluation

Four studies [21–24] evaluated their programmes descriptively with the use of questionnaires. Three of these [21–23] obtained both quantitative and qualitative data. One study [24] obtained only quantitative data. The fifth [25] study obtained purely qualitative data through interviews.

### Description and quality assessment of studies included.

The first study by Singh et al. [23], aimed to evaluate the new near-peer mentoring programme at the authors’ institution. It also compared the difference between the rate and quality of contact of mentees between their near-peer mentors and faculty mentors who were previously the only institution-recognised mentors available prior to the start of the new near-peer mentor programme. The aim of the mentoring programme at this institution was to facilitate an easy adaptation of the first years into the medical school. The near-peers consisted of students in their 2nd to 5th years. An open-ended questionnaire was sent out to all involved parties at the end of the year. The study assessed both the mentees’, near-peer mentors, and faculty mentors’ knowledge, perception and attitudes towards the institution’s mentoring programme. It also asked questions pertaining to the benefits of the mentoring programme. The key finding was that the mentees met with their near-peers more often than the faculty mentors. The reasons for this include: they were less intimidated by them, they felt they could relate better to them, and the near-peers understood them better as they had recently gone through situations they (the mentees) were now facing. The benefits identified from the study were addressed in 2 folds: to the mentees, and to the near-peers. The mentees’ benefits related to different areas including: social benefits in helping to integrate into the new environment; professional benefits: they learnt medical etiquette; academic benefits: they learnt the ‘tricks of the trade’; emotional benefits: mentors helped boost their morale, acted as stress relievers and helped them to settle down. Top benefits reported by the near-peer mentors include an improvement in problem-solving skills, responsibility and communication skills.

One of the stated aims of the study by Singh et al. [23], was to measure the quality of contact between the mentors and the mentees, however, this wasn’t addressed or reported in the study. The questionnaire used was drawn from 3 different sources, which could have led to a compromise in the validity and reliability of the new instrument created [18]. It wasn’t piloted and the means of its distribution also wasn’t stated. The study had a 50% response rate and response bias could also have affected the quality of this study- the personality of responders could have positively affected the results, however this was noted by the authors as a limitation of the study. Furthermore it wasn’t stated if the data was collected in anonymity. Another limitation was that only a single cohort of mentees, near-peer mentors and faculty mentors were surveyed. Using the CCAT, the quality score of this study was 63%.

The second study by Yusoff et al. [24]., aimed to evaluate medical student’s perceptions and attitudes toward the near-peer mentoring programme at their institution. The near-peer mentoring programme at this medical school was part of a wider programme in helping to develop student’s professionalism. The aim of this mentoring programme was to facilitate the 1st year students’ adjustment to new campus life as well as to promote their personal development. A validated questionnaire was distributed among all the 1st and 2nd years. The questionnaire collected data on demographics, knowledge, perception and attitudes towards the programme. Data on student’s perceptions and attitudes were obtained by a 5 point Likert scale. The key findings from this study pertained to the students having positive perceptions and attitudes towards the programme. They perceived it helped them reduce stress and adjust to campus life. They also reported it, as beneficial in helping to develop teamwork skills, respect and increase their self-confidence. Furthermore, they perceived it as helping to develop personal and professional qualities like accountability, responsibility, leadership skills, self-awareness, resilience, time management and punctuality. A negative significant finding identified from the study, noted was that the medical students weren’t clear about the aims of this programme. Interestingly, the proportion of female students perceiving the programme as successful was significantly higher than the male students. This paper had clearly identified aims and used an appropriate research design. It had a good response rate of 70%. It had a logical flow and the findings were well discussed. The questionnaire used to collect data had a reported Cronbach’s alpha of 0.72, 0.93 and 0.97 for knowledge, perceptions and attitude respectively. On the other hand, there was no mention of the issues of confidentiality and how the questionnaire was administered. Furthermore the paper failed to discuss the study’s limitations and the generalisability of the study. On the CCAT, this paper scored a total of 26 giving it a corresponding percentage of 65%.

The third study, McLean [22] aimed to evaluate whether studying different curricula affected peer mentoring. The aim of the mentoring programme was to assist in the social, academic and psychological integration of new students into the school. Although respondents at one point were mentored by 5th year medical students, it was included in our review, because it also captured the respondents’ experiences of mentoring while they served as mentors in their 2nd year to new 1st years. In the study, an open-ended survey was sent to 2nd year student mentors who themselves had been mentored the previous year by 5th year traditional curriculum mentors. The survey was divided into 2 parts. The first part gathered information on their experience of being mentored by 5th year traditional curriculum students whilst they were in their 1st year undergoing a problem-based learning (PBL) course. The second part gathered information on their current experience as being mentors to 1st year PBL students whilst in their 2nd year. The study found that most of the students were of the opinion that the difference in curriculum affected the mentoring process. The reasons for this include: firstly, the 5th year traditional curriculum mentors did not understand the problems faced by the 1st year PBL students and as a result could not fully empathise with them. Secondly, the two student cohorts did not share the same experiences. This was confirmed by the senior traditional curriculum members. Nonetheless they experienced friendship with their mentors, perceived the year had been made easier by knowing someone was responsible for them and they learnt some “tricks of the trade” from their mentors. As regards their mentoring experience, being mentors themselves the following year, most of the 2nd year students agreed the mentoring process was improved as a result of being able to share the same experiences and understand the issues of the new curriculum. Furthermore as mentors, they reported rewarding experiences in the affective domain. They were pleased to know that they had been able to help, knowing their mentees were doing well academically, developing friendships with their mentees, and knowing their mentees were doing well socially. Other rewarding experiences include gaining confidence in interacting with people and being encouraged to revise their schoolwork. This study had clear aims, with a detailed background leading to the development of the research question. The aims of this study were met and the study was logical and logically presented. The methods used were appropriate and the results were adequately discussed. However the survey was sent to a single cohort of student mentors- 20, out of which only 13 (65%) responded to the first part and 16 (80%) responded to the second part. The size of this cohort generates problems for the generalisability of this study. Also as with the above studies, this study surveyed a single cohort of students. Analysis using the CCAT showed that this paper scored a total of 24 giving it a corresponding percentage of 60%.

The fourth study by Kosoko-Lasaki et al. [21], .evaluated a tiered mentoring programme for minority students at their university. The aim of the mentoring programme was to increase the opportunities for counselling, mentoring and group support of the minority student population, and the study aimed to determine the outcome measures of the student mentoring programme. An assessment form was sent to all participants, which collected information on the participant’s perception and attitude towards the programme. It also collected information on the benefits of having a mentor, and a description of the mentoring relationship. This form was an 11 item questionnaire with no reports on its validation. The key findings were that mentees reported an increase in their perceived levels of professionalism. They also reported ‘camaraderie, friendship and interaction’ as the major benefits of the programme. This programme was multi-tiered with faculty members and staff mentoring the senior students. Also, the senior students were expected to mentor the freshmen or first-year students. These students go on to mentor undergraduate students hoping to study medicine post-graduation (medicine is typically studied as a graduate programme in the USA). Furthermore the undergraduate students were expected to mentor high school students with an interest in a medical degree. The limitations of this study were that the mentee experiences were captured by an un-validated assessment form. The total number of responders in this study was 19 students, out of 30 giving a response rate of 63%. Furthermore, student demographics were not provided. As a result the years/ levels of the students that participated in the study could not be ascertained. Analysis using the CCAT on this paper scored it a total of 22 giving a corresponding percentage of 55%.

The fifth and final study by Abdolalizadeh et al., [25] aimed at exploring the perceptions of mentees and mentors about the dual mentoring programme for the first-year medical students. The aim of the mentoring programme was to assist the new incoming first-years in settling in, as well as adjusting to the new outcome-based curriculum. Thirty six first-years were randomly chosen to be mentees, while 6 mentors each were selected from the 2nd and 3rd years. Each mentoring group had 6 mentees as well as a 2nd year and 3rd year mentor. The meeting times were at least once a week at the start of the programme; this reduced to 1 in 3 weeks at the end. The mentors communicated via telephone, emails and face-to-face individually or in the group. The programme was evaluated qualitatively at the end of the year using focus groups. Twenty one mentees and the 12 mentors participated in the focus group discussions. The interviews were transcribed, analysed and coded. The study identified that the mentees felt supported through having positive relationships with their mentors. They also identified appreciating having 2 mentors in different years. The mentors felt that the programme had helped to increase their personal development and awareness as well as social skills. The limitation of this study was that there was no discussion of how the questions used during the interviews were developed, and no mention of its validity. The paper had a total score of 22 giving it 55% on the CCAT.

### Outcomes

Across the five papers, similar outcomes were identified from the evaluation of the peer- mentoring programmes, which include:

#### Personal and professional development

In the Yusoff et al. [24] study, 72.5% of the mentees reported significant improvements in their team-working skills. 61.2% of the mentees felt that they had learnt respect towards themselves and their peer mentors. 58% felt they were more accountable and responsible towards their schoolwork. Other significant findings include an increased ability to solve and deal with problems, ability to make use of opportunities and an increase in their ability to serve as leaders. Furthermore, there were also significant improvements in their levels of self-awareness and self-confidence, punctuality, and time management.

Kosoko-Lasaki et al. [21] report that 100% of the mentees perceived that the mentoring programme helped them to increase in their levels of professionalism. In the Singh et al. [23] study, the mentees responded that they learnt medical etiquette from the mentoring programme. They also learnt “tricks of the trade” and how to solve academic problems. Similarly, 7.7% of the mentees in the McLean [22] study reported the mentoring programme had helped them learn the “tricks of the trade”. Qualitative responses in the Abdolalizadeh et al. [25] study also showed that the mentees perceived positive changes in their knowledge and attitude towards medical ethics and professionalism.

Four [22–25] of the five papers indicated that the peer-mentors also experienced positive behavioural changes as a result of the mentoring experience. They reported a development in their reflective, communication, and leadership skills. They also perceived improvements in their level of responsibility and problem-solving skills. Other benefits they reported include becoming more empathetic, being able to teach, and being more conscious about time-management.

#### Transitioning

In the Yusoff et al. [24] study, 42% of the medical students reported that the mentoring programme had helped them to adjust to campus life. A little over 30% of the mentors in the McLean [22] paper retrospectively reported that as mentees, the peer-mentoring programme had made the year easier knowing ‘someone was responsible for you’ by ‘meeting a wonderful person’. According to the Singh et al. [23] paper, 24% of the medical students reported that the mentoring programme had helped them to settle in. The mentees in the Abdolalizadeh et al. [25] study agreed that the psychosocial support provided by the mentors helped in the transition phase into medical school.

#### Stress reduction

Mentees in the Singh et al. [23], study reported that the near-peer mentoring programme had a ‘de-stressing and morale building’ effect on them. In the study by Yusoff et al. [24], 43% of the mentees reported that they had experienced a reduction in stress, while just under 32% reported that they developed resilience as a result of the mentoring programme. Mentees in the Abdolalizadeh et al., [25] study reported that the mentoring programme helped them reduce stress, cope with new situations, and confront difficulties.

## Discussion

Four studies [21–24] evaluated their programmes with questionnaires (Table 1). Using the Kirkpatrick’s [26] 4-level framework for evaluation, the five [21–25] studies evaluated perceived positive changes in the mentees (Level-1). None of the studies sought to measure the degree to which the participants had acquired the intended knowledge, skills, attitude, confidence and commitment based on their participation in the mentoring programme (Level-2). None also evaluated objectively for positive behavioural changes in the mentees (Level-3); or the degree to which targeted outcomes had occurred as a result of the programme (Level-4). As a result, the quality of the evaluation methods are low-moderate. This was also reflected in the Crowe Critical Appraisal Tool scores used to evaluate the quality of the studies which ranged from 55 to 65% although the CCAT tool does not map the Kirkpatrick’s 4-level framework for program evaluation. For higher levels of evaluation, perhaps the use of randomised controlled studies comparing a peer-mentored group with a group without peer-mentoring should be considered. Also, longitudinal studies following participants of peer-mentoring may be carried out to see whether acquired positive behaviour and attitude persist. Admittedly, this may be difficult and incur expenses; and researchers would also have to account for the Hawthorne effect [27]- where people modify behaviour when under observation. It would nonetheless be worthwhile as it would be interesting to objectively measure the effect of a peer-mentoring relationship on long-term personal and professional development.

The personal and professional development of mentees has been associated with the psychosocial function of mentoring [1, 28]. It helps the mentees to learn the ropes, and prepares them for upward advancement in their organisation [1, 28]. The explanation for the development of these qualities has roots in Bandura’s Social Learning Theory [29], and Vygotsky’s Social Development Theory [30, 31]. Bandura [29] postulates most learning results from modelling, and occurs as a result of the observations of actions and conduct of individuals in the environment. The observed behaviour is coded and later serves as a guide for action. Similar to this, Vygotsky [30, 31] postulates that learning is a social construct and behaviours are learnt through interaction. The development of transferable skills and characteristics in the peer mentors has been thought to be due to the process of mentoring facilitating in them a commitment to professional growth as they help their peers [32].

Transition challenges are varied. First-year students experience a change in teaching methods in the university. Some of them move to new towns or cities. Some of them move without family or friends. If these changes, potential challenges and transitions are not supported effectively, they may lead to issues of homesickness, loneliness and stress [33, 34]. Combined with these challenges, is the fact that medical education is inherently stressful and demanding [35]. It has been estimated that the amount of new material medical students in their first year are exposed to, is equivalent to learning a new language [36]. It has been shown that proper transitioning of first-years leads to better integration which in turn leads to progression and retention [5, 6].

Closely linked to transitioning is the issue of stress. None of the studies attempted to measure levels of stress both pre and post-intervention. Stress for first-years may be related to their academics. Medical students are traditionally known to be high achievers [37–39]. Often times the valuation of their personal brightness and intelligence is first put to the test during their first-year in medical school. If their effort is not in cognisance with outcomes or results, these first-year students experience a drop in self-worth and esteem [37, 40]. Stress may also arise from social factors like trying to adjust, and they may experience isolation and culture shock of the new school [4, 41]. Forming new friendships with the breaking of old ones is another common source of stress [42]. Problems with managing money, and also the combination of part-time work with their studies may also impact them negatively [33, 41]. Stress management is important because poor coping capabilities may result in later years in fitness to practice issues [43, 44]. The mental health status of first-year medical students has an important role in maintaining professionalism [34, 44, 45]. A number of studies have shown that medical students, when compared with their contemporaries in other courses, experience higher levels of anxiety, distress and burnout [34, 38]. They have shown that the prevalence of depression and anxiety in medical students when compared to the general population is high [34, 44]. It has also been reported that there is a higher rate of depression among physicians than in the general population, and it is believed that this depression begins from medical school [34, 46]^.^ In addition, medical students perceive themselves more likely to become ill than others [47]. However, as highlighted in these five studies, the provision of an effective peer mentoring scheme may help to address these issues.

## Conclusion

The sparseness of the literature for peer mentoring for medical students as indicated by the number of studies reviewed in this paper is a limitation to this study. Most of the published papers on mentoring programmes for medical students involved faculty members as mentors. Another limitation was that the studies were descriptive cross-sectional studies which did not provide much information on how near-peer mentoring facilitates its outcomes.

Peer mentorship has been referred to as a retention and enrichment scheme for higher education [48]. It has also been seen as a valuable resource in providing social and academic support to new students. Outcome measurements identified generally in higher education have focussed on objective outcomes such as retention, grade-point averages to subjective ones such as satisfaction or a reduction in stress [28].

For future research, randomised controlled studies should be carried out comparing a peer-mentored group with a group without peer-mentoring. This prevented from examining the true benefits of near-peer mentoring in helping to facilitate transition and a reduction in stress levels.

Future evaluation tools should ideally be modelled along the lines of the Kirkpatrick levels and include longitudinal follow-up for the cohort following participants of peer mentoring to see if acquired positive behaviours and attitudes persist.

In conclusion, although it is thought that students benefit from peer mentoring, little has been published in the medical literature to determine the beneficial outcomes of such schemes and clarification of what constitutes successful mentoring programmes for medical students. This systematic review has evaluated the available published literature and provides a useful basis for future studies to determine the factors involved in effective peer mentoring programmes and the scope of the outcomes in supporting success.

## Abbreviations

CCAT: Crowe Critical Appraisal Tool
ERIC: Education Resources Information Center
PBL: Problem-Based Learning
PRISMA: Preferred Reporting Items for Systematic Reviews and Meta-Analyses

## References

[1] Kram K (1985). Mentoring at work.

[2] Andrews J, Clark R. How peer mentoring enhances student success in higher education: Engineering Education Research Group. Aston University, Birmingham, 2011. https://www.heacademy.ac.uk/system/files/aston_what_works_final_report_1.pdf. Accessed 21 Mar 2015

[3] Quesnel M, King M, Guilcher S, Evans C (2012). The knowledge, attitudes, and practices of Canadian master of physical therapy students regarding peer mentorship. Physiother Can.

[4] Krause K, Hartley R, James R, McInnis C (2005). The first year experience in Australian universities: findings from a decade of National Studies.

[5] Harvey L, Drew S, Smith M (2006). The first-year experience: a review of literature for the Higher Education Academy.

[6] Collings R, Swanson V, Watkins R (2014). The impact of peer mentoring on levels of student wellbeing, integration and retention: a controlled comparative evaluation of residential students in UK higher education. R High Educ.

[7] Bulte C, Betts A, Garner K, Durning S (2007). Student teaching: views of student near-peer teachers and learners. Med Teach.

[8] Ragins B, Kram K, Ragins B, Kram K (2007). The roots and meaning of mentoring. The handbook of mentoring at work: theory, research, and practice.

[9] Lopez N, Johnson S, Black N. Does peer mentoring work? Dental students assess its benefits as an adaptive coping strategy. J Dent Educ. 2010;74:1197–1205. 21045224

[10] Sin JH, Pathickal SM, Li M. Establishment of a peer-mentoring program for student pharmacists. Am J Health-Syst Pharm. 2015; 10.2146/ajhp140544. 10.2146/ajhp140544 26386100

[11] Dennison S. Peer mentoring: untapped potential. J Nurs Educ. 2010; 10.3928/01484834-20100217-04. 10.3928/01484834-20100217-04 20210287

[12] Carragher J, McGaughey J. The effectiveness of peer mentoring in promoting apositive transition to higher education for first-year undergraduate students: a mixed methods systematic review protocol. Syst Rev. 2016; 10.1186/s13643-016-0245-1. 10.1186/s13643-016-0245-1 PMC4840870 27101733

[13] McMillan W. Transition to university: the role played by emotion. Eur J Dent Educ. 2013; 10.1111/eje.12026. 10.1111/eje.12026 23815694

[14] Singh S (2010). Near-peer role modeling: the fledgling scholars education paradigm. Anat Sci Educ.

[15] Aveyard H (2014). Doing a literature review in health and social care: a practical guide.

[16] Lefebvre C, Manheimer E, Glanville J, The Cochrane Collaboration (2011). Chapter 6: searching for studies. Cochrane handbook for systematic reviews of interventions version 5.1.0.

[17] Oppenheim AN (1992). Questionnaire design, interviewing and attitude measurement.

[18] Liberati A, Altman DG, Tetzlaff J. The PRISMA statement for reporting systematic reviews and meta-analyses of studies that evaluate healthcare interventions: explanation and elaboration. BMJ. 2009; 10.1136/bmj.b2700. 10.1136/bmj.b2700 PMC2714672 19622552

[19] Lincoln YS, Guba EG (1985). Naturalistic inquiry.

[20] Crowe M, Sheppard L, Campbell A. Comparison of the effects of using the Crowe critical appraisal tool versus informal appraisal in assessing health research: a randomised trial. Int J Evid Based Healthc. 2011; 10.1111/j.1744-1609.2011.00237.x. 10.1111/j.1744-1609.2011.00237.x 22093394

[21] Kosoko-Lasaki O, Sonnino R, Voytko M (2006). Mentoring for women and under-represented minority faculty and students: experience at two institutions of higher education. J Natl Med Assoc.

[22] McLean M (2007). Does the curriculum matter in peer mentoring? From mentee to mentor in problem-based learning: a unique case study. cMET.

[23] Singh S, Singh N, Dhaliwal U. Near-peer mentoring to complement faculty mentoring of first-year medical students in India. J Educ Eval Health Prof. 2014; 10.3352/jeehp.2014.11.12. 10.3352/jeehp.2014.11.12 PMC4309947 24980428

[24] Yusoff M, Rahim AL, Noor A, Yaacob N, Hussin Z. Evaluation of medical students’ perception towards the BigSib programme in the School of Medical Sciences, USM. Educ Med J. 2010; 10.5959/eimj.2.1.2010.or1.

[25] Abdolalizadeh P, Pourhassan S, Gandomkar R, Heidari F, Sohrabpour AA (2017). Dual peer mentoring program for undergraduate medical students: exploring the perceptions of mentors and mentees. Med J Islam Repub Iran.

[26] Kirkpatrick DL, Kirkpatrick JD (2006). Evaluating training programs: the four levels.

[27] Mayo E (1933). The human problems of an industrial civilization.

[28] Ragins B, Kram E (2007). The handbook of mentoring at work: theory, research and practice.

[29] Bandura A (1977). Social learning theory.

[30] Vygotsky LS (1978). Mind in society.

[31] Vygotsky LS (1962). Thought and language.

[32] Glass N, Walter R (2000). An experience of peer mentoring with student nurses: enhancement of personal and professional growth. J Nurs Educ.

[33] Messarra LC (2005). Sources of stress for first year students and their perception of the university employed support services: a case study.

[34] Dyrbye L, Thomas M, Shanafelt T (2006). Systematic review of depression, anxiety, and other indicators of psychological distress among U.S. and Canadian medical students. Acad Med.

[35] Shaikh B, Kahloon A, Kazmi M (2004). Students, stress and coping strategies: a case of Pakistani medical school. Educ Health (Abingdon).

[36] Brandt ML (2014). Transition from undergraduate school to medical school.

[37] Dweck CS (1999). Self-theories: their role in motivation, personality and development.

[38] Henning K, Ey S, Shaw D (1998). Perfectionism, the imposter phenomenon and psychological adjustment in medical, dental, nursing and pharmacy students. Med Educ.

[39] Widmar GE, Martin DC, Arendale DR (1994). Supplemental instruction: from small beginnings to a national program. Supplemental instruction: increasing achievement and retention.

[40] Getzlaf SB, Sedlacek GM, Kearney KA, Blackwell JM (1984). Two types of voluntary undergraduate attrition: an application of Tinto’s model. Res High Educ.

[41] McMillan W (2013). Transition to university: the role played by emotion. Eur J Dent Educ.

[42] Greenberg J (2004). Comprehensive stress management.

[43] Chang E, Eddins-Folensbee F, Coverdale J (2012). Survey of the prevalence of burnout, stress, depression, and the use of supports by medical students at one school. Acad Psychiatr.

[44] Dyrbye L, Thomas M, Massie F, Power D, Eacker A, Harper W (2008). Burnout and suicidal ideation among U.S. medical students. Ann Intern Med.

[45] Dyrbye L, Massie FJ, Eacker A (2010). Relationship between burnout and professional conduct and attitudes among U.S. medical students. JAMA.

[46] Schernhammer E (2005). Taking their own lives- the high rate of physician suicide. N Engl J Med.

[47] Raj RS, Simpson CS, Hopman WM, Singer MA (2000). Health related quality of life among final-year medical students. Can Med Assoc J.

[48] Heathcote E, Taylor P (2007). The potential contribution of change management literature to understand and support student transitions.

