# Correction to: A systematic review of the literature describing the outcomes of near-peer mentoring programs for first year medical students

**DOI:** 10.1186/s12909-018-1273-4

**Published:** 2018-07-13

**Authors:** Olawunmi Akinla, Pamela Hagan, William Atiomo

**Affiliations:** 1 Macclesfield District General Hospital, Macclesfield, UK; 2 School of Medicine, The University of Nottingham, Nottingham, UK; 3 School of Medicine, Queens Medical Centre, The University of Nottingham, Derby Road, Nottingham, NG7 2UH UK

## Correction

Following publication of the original article [1], the author reported incorrect referencing in Table 1 as the references of the papers in the table don’t match with the text.

The correct referencing for Table 1 is as folows:

Abdolalizadeh 25

Singh 23

Yusoff 24

McLean 22

Kososko-Lasiki 21
